# Are UK care homes ready for the telemedicine revolution?

**DOI:** 10.1192/bjb.2020.93

**Published:** 2020-10

**Authors:** Meta McGee, Claire Potter, Joseph Kane

**Affiliations:** ST4 Old Age Psychiatry, Belfast Health & Social Care Trust, Northern Ireland; Academic Clinical Fellow, Centre for Public Health, Queen's University Belfast, Northern Ireland; Academic Clinical Lecturer, Centre for Public Health, Queen's University Belfast, Northern Ireland; email: joseph.kane@qub.ac.uk

The restrictions implemented by coronavirus disease 2019 (COVID-19) have reawakened discussion surrounding the use of telemedicine in routine clinical practice.^1^ Nursing home residents have emerged as a particularly vulnerable group, not only with respect to the virus itself, but to the effects of social distancing^2^ and disruption to the services in place to support them. In working to stop the spread of COVID-19 within their facilities, many homes have found it difficult to sustain the non-pharmacological mainstays of management of delirium and behavioural and psychological symptoms of dementia.^2^ Telemedicine, a proposed solution to these difficulties, has been demonstrated to be both valid and acceptable to patients with dementia and care home staff^3^ but it does not appear to be part of routine practice in the UK.^4^

We therefore aimed to determine nursing homes’ capacity and enthusiasm for telepsychiatry assessments. Over a 2-week period in June 2020, we contacted senior staff at the 70 nursing and ‘elderly mentally infirm’ homes falling within the Belfast Health & Social Care catchment area and administered a short survey via telephone. Two questions; ‘how would you rate your facilities' current capacity to participate in mental health assessments via video link?’ and ‘how interested would you be in establishing the capacity to participate in mental health assessments via video link?’, were answered using a five-point Likert scale.

Participating nursing homes (56/70; 80%) reported that reliable WiFi connections and appropriate equipment (such as a tablet device, or desktop or laptop computer with webcam) were available in 41/56 (73%) and 40/56 (71%) facilities, respectively. Staff at 21/56 (38%) reported that they already felt they had the capacity to facilitate such consultations (answering ‘5’ on the Likert scale); 16/56 (29%) felt they had little (5/56; 9%) or no capacity (11/56: 20%) to currently do so. Nursing home staff answering ‘3’ (10/56; 18%) or ‘4’ (9/56; 16%) on the Likert scale indicated some capacity to participate in remote consultations, although they felt they would not be able to do so on a routine basis. Respondents were ‘very interested’ in establishing capacity to use remote consultations in 44/56 (79%) of surveys.

Most nursing homes possess the appropriate equipment to facilitate telemedicine, and the majority (84%) demonstrate an enthusiasm for doing so. The failure to adopt telepsychiatry may therefore be more closely related to factors within mental health services, such as our access to appropriate equipment, than those within nursing homes. A survey of American psychiatrists working in nursing homes reported widespread support for telemedicine, but only 13% felt they had access to appropriate equipment.^5^ It would appear, however, that obstacles to telemedicine go beyond hardware; in spite of most respondents’ access to equipment, we observed considerable variation in their perceived capacity to engage with remote consultations, perhaps suggesting a lack of comfort or familiarity with the medium. We suggest that before telemedicine becomes part of, as has been suggested, ‘the new normal’, that more detailed exploration is conducted regarding the attitudes and skills of professionals on both sides of the webcam.
